# *Revolver* and *Superior*: Novel Transposon-Like Gene Families of the Plant Kingdom

**DOI:** 10.2174/138920210790217954

**Published:** 2010-03

**Authors:** Motonori Tomita

**Affiliations:** Molecular Genetics Laboratory, Faculty of Agriculture, Tottori University, Tottori 680-8553, Japan

**Keywords:** Transposon, gene family, Revolver, Superior, structural diversity, quantitative change, evolution, plant kingdom.

## Abstract

High-throughput sequencing of eukaryotic genomes has revived interest in the structure and function of repetitive genomic sequences, previously referred to as junk DNA. Repetitive sequences, including transposable elements, are now believed to play a significant role in genomic differentiation and evolution. Some are also expressed as regulatory noncoding RNAs. Vast DNA databases exist for higher eukaryotes; however, with the exception of homologues of known repetitive-sequence-families and transposable elements, most repetitive elements still need to be annotated. *Revolver* and *Superior*, both discovered in the Triticeae, are novel classes of transposon-like genes and major components of large cereal genomes. *Revolver*  was isolated from rye *via*  genome subtraction of sequences common to rye and wheat. *Superior*  was isolated from rye by cleavage with *Eco*O109I, the recognition sites of which consist of a 5′- PuGGNCCPy-3′ multi-sequence. *Revolver*  is 2929–3041 bp long with an inverted repeat sequence on each end. The *Superior*  family elements are 1292–1432 bp in length, with divergent 5′ regions, indicating the presence of considerable structural diversity. *Revolver*  and *Superior*  are transcriptionally active elements; *Revolver*  harbors a single gene consisting of three exons and two introns, encoding a protein of 139 amino acid residues. *Revolver*  variants range in size from 2665 bp to 4269 bp, with some variants lacking the 5′ region, indicating structural diversity around the first exon. *Revolver*  and *Superior*  are dispersed across all seven chromosomes of rye. *Revolver*  has existed since the diploid progenitor of wheat, and has been amplified or lost in several species during the evolution of the Triticeae. This article reviews the recently discovered *Revolver*  and *Superior*  families of plant transposons, which do not share identity with any known autonomous transposable elements or repetitive elements from any living species.

## PLANT TRANSPOSABLE ELEMENTS

In higher eukaryotes, genes required for cellular function can comprise as little as 20% of the genome [1, 2], and occur in islands separated by repetitive DNA sequences [3, 4]. These repetitive regions comprise >70% of the genomes and are often referred to as junk DNA. With the advent of high-throughput DNA sequencing, it has become apparent that transposable elements constitute a large proportion of the repetitive DNA component of most eukaryote genomes, that is, at least 45% of the human genome [5], and 50–80% of some grass genomes [6, 7]. Advances of eukaryotic genomics have revived interest in the structure and function of repetitive genomic sequences. Amplification and rearrangement of repetitive sequences, including transposable elements, are now believed to play a significant role in genomic differentiation and evolution [8, 9]. Moreover, a substantial proportion of the genome is expressed as regulatory noncoding RNAs, some of which are reconstructed from transposable elements [10-12].

Transposable elements are divided into two major classes according to their mode of transposition [13]. Class I elements can replicate by transcription of the genomic element, followed by reverse transcription of the RNA to generate a cDNA copy, which is then integrated back into the genome [14]. A representative class I element is the long terminal repeat (LTR) retrotransposon, which has both structure and life-cycle similar to elements in retroviruses [15-17]. LTR retrotransposons are ancient and ubiquitous, and are major components of plant genomes [17-21]. They are believed to be significant contributors to plant genome evolution because their replication strategy has the potential to result in an explosive increase in copy number and for insertional genomic change [9, 22].

Class II transposable elements move by excision from one chromosomal location and reintegration elsewhere in the genome. They are distinguished by terminal inverted repeats (TIRs) and are divided into three superfamilies – *hAT* , CACTA and MULU – on the basis of the homology of the TIRs and the transposase genes carrying out the cut-and-paste process. Transposons are widespread in plants [23, 24], but are generally quite low in copy numbers, ranging from tens to hundreds per genome. However, an analysis of the growing genomic DNA sequences databases has revealed that plant genomes can also harbor up to 30,000 copies of miniature inverted-repeat terminal elements (MITEs), which are classified as class II elements [25]. MITEs, along with LTR retrotransposons, have made important contributions to the evolution of plant genome organization [26, 27].

Widely distributed transposable elements are the most rapidly evolving fraction of the eukaryotic genome [28], because the methylated and heterochromatic state of most highly repetitive elements is more susceptible to sequence change than gene-coding sequences [29, 30]. In general, the genomes of higher eukaryotes contain thousands, even millions, of seemingly inactive transposable elements, which may be a source of interspecific sequence divergence. Species-specific repetitive elements serve as genetic tools for developing DNA markers dispersed throughout the genome [31]. As well as the development of molecular markers, active transposable elements have been significant tools in functional genomics studies. Their incorporation into the host genome enables insertional gene mutagenesis and gene tagging. In rice, for example, the LTR retrotransposon Tos17 is used to generate mutant lines [32]. Class II *Ac* (*hAT*) and *Mutator* (MULU) transposons have also been widely used for gene cloning [33].

Despite the vast DNA databases that exist for higher eukaryotes, most repetitive components of genomes are yet to be identified and annotated; only elements homologous to known repetitive-sequence-family and transposable elements or their derivatives are annotated [34, 35]. Efforts have been made to identify novel active genomic components that might be useful molecular tools. Among the Triticeae tribe, rye (*Secale cereale*) has been used in wheat (*Triticum aestivum* L.) and triticale breeding programs, as a source of genes conferring agronomically important traits such as stress resistance. The rye genome has a 1C DNA content of 3.9 Gb – the highest among the Triticeae – and Cot analysis estimates that 24% of the rye genome is rye-specific [1]. Repetitive sequences comprise 92% of the genome [36], and. rye-specific repetitive sequences have been useful molecular probes for the determination of introgressed genomes and the genomic constitution of wheat–rye hybrids [37, 38]. In the rye genome, dozens of known transposable elements that are classed as Ty1-*copia* elements [39, 40], Ty3-*gypsy* elements, LINE elements, SINE elements, CACTA elements, or MITE elements have been registered in the GenBank database (http://www.ncbi.nlm.nih.gov/Genbank/index.html). However, for a long time, active transposable elements were not identified in rye, wheat or wheat relatives. One reason for this may be that repetitive sequences were isolated mainly from relic DNA, which is not susceptible to methylation-sensitive restriction enzymes used to isolate repetitive elements [41-43].

Recently, the first active plant foldback (FB) transposon *RYS1* was identified [44]. Two new classes of transposon-like genes, *Revolver* [45] and *Superior* [46], have also been identified in rye, from repetitive non-methylated DNA [46, 47]. These novel genomic components of the huge and complex rye genome are distinct from known class I and class II transposable elements, and might be useful as tools for gaining insight into genome structure and evolution in the Triticeae, or for use in molecular breeding programs. The following sections describe the identification and characterization of *Revolver* and *Superior* elements.

## ISOLATION OF NOVEL REPETITIVE ELEMENTS INCLUDING REVOLVER BY GENOMIC SUBTRACTION

Novel repetitive DNA elements have been successfully identified through a process of genomic subtraction between wheat and a rye-chromosome wheat addition line to identify rye-specific sequences [47]. The authors used the bread wheat (*Triticum aestivum* L.) cultivar Chinese spring, and a rye-chromosome Chinese spring wheat addition line, which carries the 6R chromosome from self-fertile rye (*Secale cereale* L.). Repetitive sequences from wheat have evolved and differentiated by combining units of different repetitive sequences [41, 48-51]. To obtain minimum repetitive-sequence units, the authors of the study digested genomic DNA from the rye chromosome-addition wheat into fragments ≤2 kb using the four-base cutting restriction enzyme *Mbo*I. A DNA library was then established, in which the sequences common to wheat were subtracted from the rye-chromosome-addition wheat genome. To achieve this, randomly cleaved genomic DNA from wheat was generated by sonication. This DNA was mixed in excess with the restricted products of rye-addition wheat, and the mixture was denatured and then re-annealed. In the process of re-annealing, the rye *Mbo*I fragments with sequences in common with wheat DNA annealed with the excessive amounts of wheat fragments of different lengths and terminal forms, whereas the *Mbo*I fragments with repetitive sequences specific to rye re-annealed to restore their original cohesive terminals. Using this approach, only rye-specific double strands with cohesive ends could be ligated into the subtraction library vectors. The deletion-enrichment scheme was first described for obtaining mouse Y-chromosome genes [52], and had not been reported for plant genomes. Using genomic subtraction, the authors successfully isolated rye-specific DNA elements [47].

A total of 77 recombinant clones were isolated in this study, which represented 1.2% of those generated in the control experiment using the shotgun method to clone *Mbo*I fragments that had not undergone subtraction. Thus, it appears that 98.8% of the *Mbo*I fragments from the rye-chromosome wheat addition line annealed with the sonicated wheat fragments, whereas 1.2% of the *Mbo*I fragments were potentially rye-specific.

From this library, the 77 plasmid DNAs were screened by differential dot hybridization against rye and wheat genomic DNA. Of these, 14 clones were rye-specific, hybridizing strongly to rye DNA but not to wheat DNA. Seven of these clones were identified as belonging to the tandem 350 bp family [41, 42], and one clone to the dispersed R173 family [53, 54], both of which are rye-specific repetitive sequences. Additionally, a single clone contained an 89 bp unknown sequence (GenBank accession AB304276). These results indicate that the subtraction method to remove sequences common to wheat and rye was effective and led to the successful identification of a novel 89 bp sequence from rye, which was further characterized.

## STRUCTURE OF DISPERSED TRANSPOSON-LIKE GENE REVOLVER

Southern blot analysis confirmed that the 89 bp rye sequence identified through genome subtraction was present in the genomes of *Secale cereale*, *Secale vavilovii* and *Secale montanum*, but not in the wheat genome [45, 47]. Therefore, the 89 bp segment is derived from a repetitive sequence found specifically in the R genome of the genus *Secale*. In order to determine the full-length sequence of the repetitive element harboring the 89 bp fragment, a rye genomic library was constructed and screened with the 89 bp probe [45]. The plaque hybridization analysis found approximately 800 positive plaques from the rye genomic DNA library. Six positive clones were chosen at random, and the restriction mapping revealed that each clone was derived from a different area of the rye genome.

Three clones were sequenced in full, and two that were 92% identical, contained complete structure of the repetitive element harboring the 89 bp segment: 3041 bp long in one and 2929 bp long in the other (GenBank accessions AB124639–124640) [45]. The regions flanking the 3 kb elements did not show any homology between the two clones. Analysis revealed that the consensus sequence of the element contained 20 bp of incomplete TIRs on both ends (5'-TGTgAcGCCCgaGAccGACg-3', 5'-TGTaAtGCCCagGA tggGAC-3'), and sub-terminal short repeat sequences on the 5' end (5'-TCCAGAAGAT-3'). A fluorescence *in situ* hybridization (FISH) experiment using the 3041 bp clone as a probe showed that the 3 kb insertion is dispersed across the seven chromosomes of rye [45]. 

Despite previous extensive characterization of the repetitive elements in the rye genome, the 3 kb element did not show similarity to any known rye repetitive element: the 350 bp family [41, 42], the 120 bp family [41, 55], the 5.3H3 family [43], the R173 family [53, 54] or pSc250 [56]. The entire structure of the 3 kb transposon-like element was also distinct from class I and class II transposable elements. This novel transposon-like element was named *Revolver* [45].

## CHARACTERIZATION OF THE REVOLVER mRNA

**Fig. (1):**
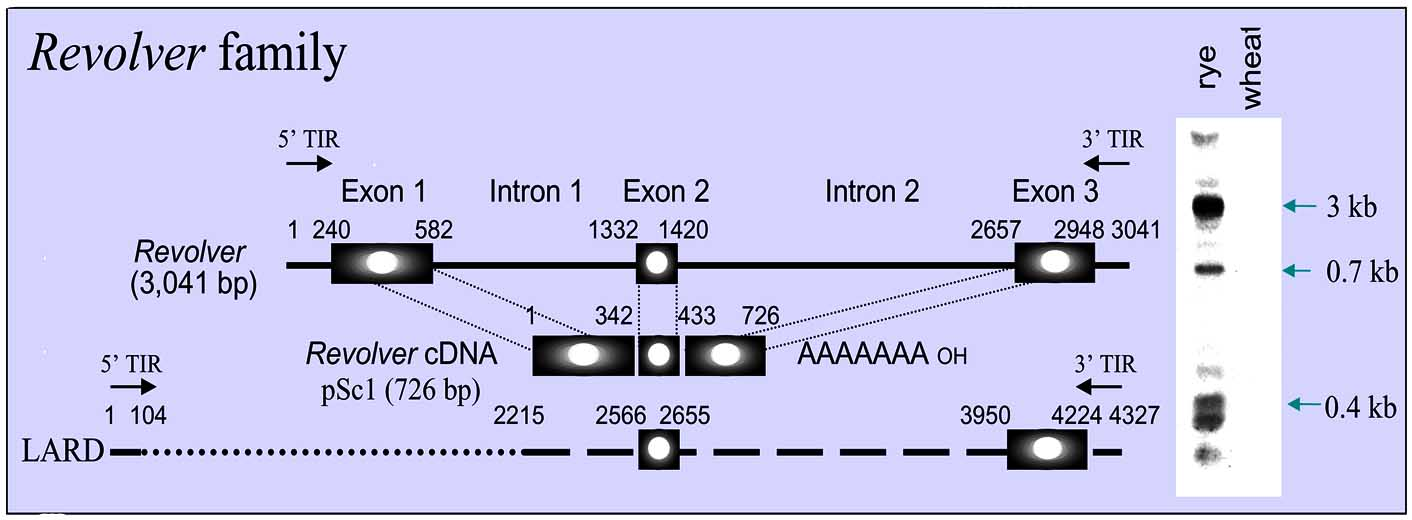
Structure of Revolver. Revolver consists of 3 exons and 2 introns. However, members of the *Revolver* family showed structural variation in the 5' region, especially in length of 1^st^ exon and 1^st^ intron. These variants of *Revolver* family yielded several sizes of transcripts in rye but no Northern blots in wheat.

*Revolver* is expressed extensively in rye, but there is no transcript in wheat [45]. Weak transcripts are also detected in a rye-chromosome wheat translocation line [45]. A *Revolver* cDNA clone was identified from a leaf cDNA library, which is 80% identical to the 3 kb genomic probe (AB124665). The full-length cDNA of *Revolver* is 728 bp long (AB124666). Nucleotide sequence comparison between the two original 3 kb *Revolver* genomic DNA clones and the cDNAs revealed that the *Revolver* element consists of three exons of 342 bp, 88 bp and 292 bp, and two introns of 750 bp and 1237 bp (Fig. **1**). Splice acceptor and donor sites are also evident at the exon–intron junctions. A putative TATA box is located at base 221, with a cap site at base 261 and a possible polyadenylation signal AATAAA at base 2918 [45]. Therefore, *Revolver* harbors a single gene consisting of three exons and the initial 89 bp clone obtained by genomic subtraction is located around the second exon.

*Revolver* cDNAs have been cloned from *Secale sylvestre*, *Dasypyrum villosum*, *Triticum monococcum* and *Aegilops tauschii* using 22-mer primers from each end of *Revolver* [45]. These cDNAs (AB124645, AB124666, and AB304271–AB304275) contain a single open reading frame (ORF) encoding a protein of 139 amino acid residues. The ORF does not show homology to known transposases; however, the predicted *Revolver* protein shows similarity to the AsnC/Lrp subfamily of transcriptional regulators and a glycerol-3-phosphate transporter, and includes a DDE motif. Because it has terminal inverted repeats and encodes a DNA binding-like protein, *Revolver* is considered to be a class II transposable element. However, the inverted repeat sequences and the encoding gene, which identify *Revolver* as a class II transposon, are unique sequences that are quite different from known class II transposons. Moreover, the entire structure of *Revolver* does not share whole identity with either class I or class II autonomous transposable elements.

The Ty1-*copia* group of retrotransposon comprises a major portion of plant genomes [17, 31]. *BARE*-1, one of the Ty1-*copia* retrotransposons [57, 58], is transcribed and is dispersed throughout the genome of barley (*Hordeum vulgare*) [59, 60]. *Revolver* is quite different from retrotransposons, although it shows partial homology (60%) at both end regions (5' end 123 bp, 3' end 777–2112 bp) to LTRs (long terminal repeats) of large retrotransposon derivative (LARD) elements, such as the nested insertion in the 3' LTR of *BARE*-1 (3130–4960 bp) [57]. LARD LTR (large retrotransposon derivative–long terminal repeat) is regarded as a solo LTR of the non-autonomous retrotransposon element in barley [61]. The LTRs are two sequences of 5 kb, found to be similar to this insertion in a 66 kb stretch of the barley genome [62, 61]. The LARD LTRs contain a region homologous to both 5' and 3' ends of the *Revolver* element (Fig. (**1**)). At the 5' terminus, LARD LTRs have a region of 123–149 bp homologous to upstream of the transcription initiation site of *Revolver*. At the 3' terminus, LARD LTR-1 (4960 bp) has a region of 2112 bp showing 60% homology to *Revolver*, from the middle of the first intron to the 3' terminus of *Revolver*. Another LARD LTR nested in *BARE*-1 has a region of 777 bp showing 60% homology to the middle of the second intron to downstream of the third exon of *Revolver*. Both 3' termini coincide with the 3' terminus of the untranslated region downstream of the third exon of *Revolver*. However, no homology to *Revolver* was observed in 631–2176 bp of LARD-LTR1 or 598–2353 bp of LARD-LTR nested in *BARE*-1. As for the region of about 2 kb between these end regions, LARD LTRs lack the region from the first exon to the middle of the first intron. Moreover, the central regions of the LARD LTRs are highly variable [61], but *Revolver* is entirely conserved. LARD LTRs also contain sequences not present in *Revolver* in place of the first exon, resulting in noncoding sequences. *Revolver* and LARD LTRs may be evolutionally related, but LARD LTRs are a structural part of an LTR retrotransposon, whereas *Revolver* is a single gene consisting of an exon–intron structure. A gene-encoding LTR has never been identified. Therefore, *Revolver* is quite distinct from LARD LTRs, because exon 1 and exon 2 of *Revolver* are replaced in the LARD LTRs by different sequences so that a coding region is not present and an autonomous element has never been reported. The presence of a *Revolver*-like element in barley suggests a wide prevalence of *Revolver* among the Triticeae.

The novel high-copy element *Revolver* is transcriptionally active in rye. Some of transposon-like elements exist in high copy numbers in the genomes of most eukaryotes, but the great majority of them are inactive, and only a small portion of them retain the ability to transpose [63, 64]. Very few transposons have been shown to be transcriptionally active. The *copia*-like retroelement *BARE*-1 is transcribed in somatic tissues of barley [59]. Some LTR retrotransposons, such as tobacco Tnt1, Tto1 and *BARE*-1 that are largely inactive, can be transcriptionally activated under conditions of biotic and abiotic stress, including wounding, oxidative stress, and pathogen infection [65, 66]. After stress-induced transcription, the rice LTR retrotransposon Tos17 increases in copy number in the genome [67]. In maize, a survey of more than 4 × 10^5^ expressed sequence tag (EST) sequences identified only 56 retrotransposon cDNAs, supporting the notion that most retrotransposons are inactive. Furthermore, most of these sequences are derived from the low-to-middle repetitive LTR retrotransposons, and not from the very high copy number elements that have been responsible for doubling the size of the maize genome in the past 5–6 million years. In humans, only 30–60 L1 retrotransposon elements out of 5 × 10^5^, which comprise 45% of the genome, are thought to be active [68]. In contrast to the low-level activity of high copy retrotransposons, the highly repetitive *Revolver* element is transcribed strongly and may retain mobility and mutagenetic potential.

A transcriptionally active *Revolver* gene is well conserved among the Triticeae members. The methylated and heterochromatic state of most transposons can cause them to change sequence more rapidly than genes [29, 30]. For example, regulation at any stage of the replication cycle for retrotransposons (transcription, translation, reverse transcription and integration of the cDNA) can limit the transposition. Furthermore, the paucity of maize retrotransposon-derived ESTs indicates that some epigenetic mechanisms might repress their transcription. In contrast to these silenced retrotransposons, *Revolver* is transcriptionally active and short RNA homologues causing RNAi silencing [69-71] have not been observed on Northern blots [45]. *Revolver* has not suffered from epigenetic silencing mechanisms and might have retained strong transcriptional activity during the long evolution of the Triticeae.

## ISOLATION OF THE RYE GENOME-SPECIFIC ELEMENT SUPERIOR

The *Eco*O109I restriction enzyme cleaves at recognition sites consisting of 5′-PuGGNCCPy-3′ multi-sequences, which are present at a high frequency in rye repetitive repeat families [47]. For this reason, it was used to recover repetitive DNA elements from the rye genome [46] by digesting genomic DNA of self-fertile rye to completion followed by blunting of the cleaved DNA ends. Fragments were used to establish a DNA library from the rye genome. Eighty-six recombinant clones were randomly isolated and dot-hybridization with the total DNA of rye and wheat revealed that 20 of these had strong hybridization signals only for rye.

The core units of the screened repetitive clones were analyzed by Southern blot hybridization after digestion with *Eco*O109I [46], which confirmed that five of the 20 clones were rye-specific. The clone pSc27 contained an unknown 495 bp sequence (AB464948) and did not show any similarity to known repetitive elements in rye, such as the 350 bp family, the 120 bp family, the 5.3H3 family, the R173 family, and pSc250 [41-43, 51, 53-56, 72]. To determine the entire sequence of the repetitive element harboring the 495 bp fragment, a rye genomic library was screened using the 495 bp sequence as a probe [46]. Plaque hybridization analysis found approximately 800 positive plaques. Three positive clones with insertions of 11.5 to 16.0 kb were chosen at random. Restriction mapping of the three insertions revealed that each was from a different area of the rye genome.

The clones were sequenced in full to determine the structure of the repetitive elements harboring the 495 bp segment [46]. The three full-length sequences were 1432 bp, 1324 bp, and 1292 bp long (AB464949–464951), and showed 88% identity to each other [46]. The regions flanking the repetitive elements showed no homology between each other. The consensus sequence also lacked terminal inverted repeats on both ends. One of the sequenced clones contained two instances of a homologous 192 bp region (Fig. (**2**)). Despite extensive characterization of the repetitive elements in the rye genome, these elements showed no similarity to any known rye repetitive elements [41-43, 51, 53-56, 72]. The entire structures of the elements did not share identity with class I or class II transposable elements or known repetitive elements. A search in BLASTN against all DNA databases of living organisms revealed that the repetitive elements had similarities only to several segments in Bacterial Artificial Clones (BACs) and ESTs from barley (Fig. (**2**)). These included BAC No.631P8 (DQ249273) and BAC No.745c13 (AF474071) from barley. Moreover, the 495 bp sequence (AB464948) in clone pSc27 was repeated twice inside BAC No.631P8. Three barley elements in the BACs included the complete structure of the repetitive element harboring the 495 bp segment: one in DQ249273 that was 1531 bp long, one in DQ249273 that was 1293 bp long, and one in AF474071 that was 1160 bp long, with 59% to 64% identity to each other. The presence of similar elements in barley suggests a wide prevalence of this dispersed repetitive sequence family, which has been named *Superior*, among the Triticeae.

A FISH experiment using the 495 bp sequence as a probe revealed that *Superior* is dispersed across the seven chromosomes of rye [46]. The copy number of *Superior* was calculated by slot blot hybridization, to be 1 × 10^4^ in the rye genome, but only 1 × 10^2^ in the wheat genome. If *Superior* is a mobile element, as many as 1 × 10^4^ copies of the *Superior* family have been generated and spread throughout the rye genome since the evolutionary event that separated rye and wheat. The consensus sequence of *Superior* does not contain TIRs on each end. This may be a result of the fact that *Superior* shows extreme structural diversity, especially around the 5′ region (Fig. (**2**)). The *Revolver* family also shows considerable length variation, which can be attributed to structural variations at the first exon [45]. Most transposable elements are more susceptible to sequence changes than gene-coding sequences, due to their methylated and heterochromatic state [29, 30]. Despite this, several barley ESTs showed similarity to the *Superior* repetitive element [46].

Following reports of Appels *et al*. [42, 73, 43], several repetitive DNA families from rye were cloned from methylated relic DNAs. Repetitive sequences from wheat species are though to have differentiated by combining units of different repetitive sequences regardless of methylation level [41, 48-51]. Repetitive elements have been isolated and cloned from non-methylated genomic regions using a method of genomic subtraction [47]. The sizes of the repetitive core units in the four kinds of rye-specific clones obtained were distinguished by *Eco*O109I digestion [47].

The enzyme *Eco*O109I can generate polymorphic DNA fragments of unpredictable length because it recognizes the ambiguous motif 5′-PuGGNCCPy-3′, eventually representing 16 different heptamer motifs. *Eco*O109I is insensitive to (cytosine-5)-methylation of 5′-CG-3′ sites, because none of the possible 16 different *Eco*O109I recognition motifs overlaps with this (cytosine-5)-methyltransferase recognition motif. On the other hand, when the 5′-PuGGNCCPy-3′ sequence is followed by a G in the genomic DNA, all *Eco*O109I recognition motifs overlap with two of the possible four different (cytosine-5)-methyltransferase recognition motifs of 5′-CNG-3′. Moreover, all *Eco*O109I recognition motifs, regardless of which nucleotide follows the 5′- PuGGNCCPy-3′ sequence, always overlap with eight of the 16 possible different (cytosine-5)-methyltransferase recognition motifs of 5′-CNN-3′. In the rye genome, however, only a single homologue of (cytosine-5)-methyltransferase has been localized on chromosome 6R [74] and no sequence information on (cytosine-5)-methyltransferase is available in the NCBI nucleotide or EST databases. Therefore, it is not known whether (cytosine-5)-methyltransferase activity affects the two additional motif classes 5′-CNG-3′ and 5′-CNN-3′.

Using *Eco*O109I digestion and genome subtraction, it was pertinent that all clones hybridized strongly to *Eco*O109I repetitive units of different lengths, namely, 380 bp, 960 bp, 5 kb, and 5.5 kb [47]. Total rye genomic DNA was cleaved with *Eco*O109I and different rye-specific repetitive elements were classified into three classes of rye-specific repetitive elements, based on their sequences: pSc16, pSc27, and pSc5 [46]. These clones hybridized to rye but not to wheat DNA. One well-known rye-specific family of repetitive elements, the 350 family (pSc16, pSc81, pSc109), which is organized into multimers with a 380 bp core unit, was easily recovered. Moreover, new sequence families (pSc27 and pSc5) were successfully identified among 20 clones screened from a small population of 86 clones [46]. The unique element from the pSc27 class identified was named *Superior*. *Eco*O109I could therefore be a useful tool for studying the organization and differentiation of large amounts of repetitive sequences in the rye genome.

The full-length sequence of the novel dispersed repetitive sequence family *Superior* harboring the 495 bp fragment of pSc27 was determined using a rye genomic library [46]. As mentioned earlier, the structure of the *Superior* elements do not share any identity with class I or class II transposable elements or known repetitive elements, but are similar to barley BAC sequences. The presence of similar elements in barley indicates the wide prevalence of *Superior* among the Triticeae.

Several barley EST clones also showed similarity to the *Superior* repetitive element (Fig. (**2**)). Analysis revealed that *Superior*, like *Revolver*, is transcriptionally active. This is in contrast to the methylated and silenced retrotransposon R173 family in the rye genome [54, 47]. The RNA secondary structure of *Superior*, as predicted by the software SCARNA, consists of four well developed stems with hairpin loops. The stems are predicted to have at least ten internal loops. Because SCARNA aligns the candidate sequence with known secondary stem structures, the well developed stem structure of *Superior* suggested that *Superior* has a role as RNA.

## AMPLIFICATION AND ELIMINATION OF REVOLVER AND SUPERIOR IN THE TRITICEAE GENOME

The distribution of *Revolver* in the Triticeae has been analyzed by Southern and slot blotting techniques [45]. The *Revolver* cDNA probe hybridized strongly to gel blots of the wheat relatives *Secale* sp. (RR) and *D. villosum* (VV), and moderately to the wheat ancestral species *T. monococcum* (AA), *Aegilops speltoides* (SS), *T. dicoccum* (AABB) and *Ae. tauschii* (DD). These cereals may share a large *Revolver* superfamily stemming from their common progenitor. On the contrary, *Revolver* cDNA did not hybridize to *T. aestivum* genomic DNA. DNA slot blot analysis confirmed that *Revolver* elements are abundant in the genomes of *Secale* sp. and *D.* *villosum* (over 10^4^ copies), and *Revolver* is present at moderate copy numbers in the wheat ancestral species *T. monococcum* , *T. dicoccum* and *Ae. tauschii* (3 × 10^3^ – 8 × 10^3^ copies). Bread wheat is an allohexaploid carrying three different subgenomes, A, B, and D [75] resulting from two independent hybridization events. The first combined the A genome of the wild diploid wheat *T. monococcum* (AA) and the B genome, which has the greatest genetic similarity to the S genome of *Ae. speltoides* [76-78]. This resulted in the tetraploid ancestor of modern *Triticum* species *T. dicoccum,* which hybridized with *Ae. tauschii*, the diploid donor of the D genome, over 8000 years ago, resulting in hexaploid wheat [79]. *Revolver* is very rare in bread wheat (10^3^ copies). These observations indicate that *Revolver* has existed since the diploid progenitor of wheat, and it has been amplified in rye and some other species, whereas *Revolver* has been lost from bread wheat through evolution accompanied by hexaploidization. *Revolver* exhibits quantitative variation during evolution of the Triticeae.

The rye haploid genome contains 3.9 Gb of DNA and is the largest among the Triticeae genomes. The highly repetitive *Revolver* and *Superior* elements have been visualized over the entire lengths of the rye chromosomes in FISH experiments. Generally, angiosperm genomes vary tremendously in size; some species have less than 50 Mb of DNA per haploid genome and others have more than 85,000 Mb [80]. The lack of correlation between the complexity of the organism and the size of the genome has long been recognized as a C value paradox [81]. There is great variability in genome size among the grass family Poaceae: 450 Mb for rice, 2500 Mb for maize, 5000 Mb for barley, and 16000 Mb for hexaploid wheat [82]. Most of the variation in genome size is attributable to the amount of repetitive DNA, which comprises 70% of cereal genomes [1, 3]. Cot analysis has estimated that repetitive sequences comprise 92% of the rye genome [36], and 24% of these have been differentiated in a rye-specific array [1]. The considerable accumulation of *Revolver* and *Superior* elements in rye may be one of the reasons why the rye genome has became so enlarged compared with other members of the Triticeae. As many as 2 × 10^4^ copies of the *Revolver* family and as many as 1 × 10^4^ copies of the *Superior* family have been generated and spread throughout the rye genome as mobile elements since the evolutionary event that separated rye and wheat. The considerable variation of *Revolver* copy number among the wheat-related species indicates their propagation activity during evolution of the Triticeae tribe. The name of the novel transposon-like gene *Revolver* means a dynamic factor influencing genome construction through the evolution of the Triticeae.

The structure of the *Revolver* and *Superior* elements is quite different from those of known repetitive elements. It is well known that repetitive DNA comprises 70% or more of many large plant genomes, such as those of maize, wheat, and barley [6, 7, 60, 82]. Of the repetitive DNA, the LTR retrotransposons in particular have increased copy numbers [84, 85] and have thus contributed greatly to the expansion of these genomes [5, 22, 58, 86, 87]. Over a broad range of organisms, retrotransposon copy number appears to be correlated with genome size. The small genome of the yeast *Saccharomyces cerevisiae* (13 × 10^6^ bp) contains 51 full-length retrotransposons [88]. In the large genome of maize (2500 Mb), some retrotransposons have copy numbers exceeding 2 × 10^4^ per haploid genome [6, 83, 86, 89], and all of the retrotransposons sequenced appear to have been inserted within the last 6 million years, leading to a doubling of the size of the maize genome [30]. The copy number of the DNA-type (class II) transposable element is generally less than that of the highly repetitive retrotransposons, but recent analysis of the growing genomic databases has revealed that plant genomes harbor a huge copy number of DNA-type transposable elements termed MITEs [25, 27]. Class II transposable elements such as MITE and CACTA elements may also have contributed to the expansion of plant genomes [25, 27, 90, 91]. Dozens of different transposable elements that belong to different classes identified in the rye genome have been deposited in the GenBank database. They include *Copia* elements (e.g. AJ240111; 32 clones in total), *Gypsy* elements (e.g. AJ295137), LINE elements (e.g. AB457042), SINE elements (e.g. AB046150), CACTA elements (e.g. AF492376), and MITE elements (e.g. EF077404). *Revolver* and *Superior* are clearly different from both the retrotransposons (Class I) and the DNA-type (Class II) transposable elements. They are also distinct from other retrotransposons such as SINE and LINE element, which constitute 10% of the human genome and are abundant in mammalian genomes [92-94]. The transposon-like genes *Revolver* and *Superior* are novel members of a major genomic component, as are LTR retrotransposons, SINE, MITE, and CACTA elements, which have contributed to plant genome construction and evolution.

*Revolver* is less frequent in the genome of hexaploid bread wheat than its ancestors. The polyploidy that arises either by duplication of a single genome or by the acquisition of a few genomes from related species (allopolyploidy) is a major force in the evolution of plants; 50–70% of angiosperms have experienced at least one episode of polyploidy in their history [95, 8]. The combination of A, B and D genomes into a single nucleus in wheat may have generated more incompatibility than harmony for the propagation of some genes [96-98]. *Revolver* contains a single ORF encoding a 139 amino acid residue protein, which features a transcriptional regulator able to bind DNA [45], suggesting that the *Revolver* element can transpose in a cut-and-paste fashion, like class II elements. In wheat, no transcript of the *Revolver* gene is observed by Northern blot and no *Revolver* cDNA is recovered by RT-PCR [45]. An EST homologue of *Revolver* is highly degenerated compared with cDNA sequences obtained from other wheat-related species. *Revolver* may have been eliminated from the wheat genome because of losing the gene activity needed to reintegrate into the host wheat genome after polyploidy-induced incompatibility among the three genomes.

As well as having a history of genome expansion, flowering plants appear to have undergone genome contraction [99-101]. One possible mechanism that reduces genome size is unequal intrastrand recombination between two tandem repeats in direct orientation on the same chromatid. The outcome of this type of event is a net deletion of one repeat and the sequences between the repeats. Unequal intrastrand recombination between adjacent LTR retrotransposons might lead to a decrease in the size of a genome, a phenomenon that is supported by the abundance of solo LTRs and the general absence of the LTR-internal-LTR structure [99]. Deletions of retrotransposons are relatively common in insects [102]. Highly saturated *Revolver* sequences in the wheat polyploid genome could generate potential sites for unequal intrastrand recombination, and recombination between adjacent *Revolver* elements might have reduced the copy number in the wheat genome. The considerably destructive variants of *Revolver* may have stemmed from such unequal recombination events.

Rye is well adapted to extreme climatic and soil conditions, making it a useful genetic resource for the breeding of its major cereal crop relatives. Cultivated rye (*Secale cereale* L.) has been used as a gene source for wheat and triticale breeding by interspecific chromosome introgression and rearrangement, translocation or substitution. A representative achievement of this type of manipulation is the introduction of a stem rust resistance gene into wheat. Rye also has the lowest requirements of the cereal crops for chemical treatments like fertilizers or pesticides, making it an ecologically desirable crop for specific regions such as northern Europe. The rye genome still has a gene resource potential for future improvement of wheat. Rye-specific repeated sequences have been useful as probes to analyze alien chromatins and chromosomal constitutions in wheat-rye crossbreeding [37, 38]. *Revolver* and *Superior* are enriched in the genomes of *Secale* species but are rare in the bread wheat genome. These novel rye-specific elements might serve as molecular markers for rye DNA introduced into bread wheat and other plant species. The overall dispersion along all chromosomes might not add a new usable feature for the probe compared with the already established *in situ* probes [37, 103-105]. However, *Superior* might be an effective tool for using PCR methods such as SSAP (Sequence-specific Amplified Polymorphism) [106] to develop molecular tags for rye chromosomes, because the structural diversity, especially around the 5′ region, can yield many polymorphisms, as has been shown with *Revolver* [45, 107]. *Revolver* is an effective tool for developing molecular tags for transferring useful germplasm of the wheat relatives rye and *Dasypyrum* into the wheat genome. *Revolver* has the potential to be used in FISH analyses as a Southern probe for genotyping and as a dispersed PCR entry point to amplify rye-specific multiple genomic fragments [107]. *Revolver* is attractive as an index of genomic evolution and as a landmark of chromosomes useful for evaluating evolutionary relationships among the tribe Triticeae.

In conclusion, genomic cloning by genome subtraction or by using the *Eco*O109I restriction enzyme to cut PuGGNCCPy multi-sequences has led to the discovery of *Revolver* and *Superior*, novel members of the major genomic component. Similarly to LTR retrotransposons and MITEs, *Revolver* and *Superior* occur at drastically different copy numbers between bread wheat and rye, making them a useful genetic resource for wheat breeding. *Revolver* consists of a single gene encoding a DNA-binding-like protein, which is transcriptionally active and does not suffer from epigenetic silencing. *Revolver* elements feature an autonomous transposon, which may complement *in trans* the propagation of related elements, including LARD, which might have contributed to plant genome expansion, rearrangement and evolution.

## Figures and Tables

**Fig. (2):**
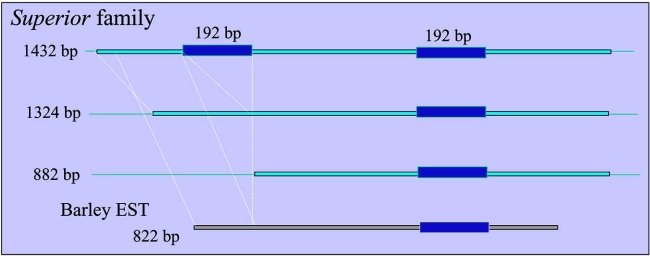
Structure of *Superior*. *Superior* elements obtained from the rye genome had structural mutations at the 5'-side. The longest sequence contained 2 instances of a homologous 192 bp region.
